# Induction of ER Stress in Macrophages of Tuberculosis Granulomas

**DOI:** 10.1371/journal.pone.0012772

**Published:** 2010-09-15

**Authors:** Tracie A. Seimon, Mi-Jeong Kim, Antje Blumenthal, Jovanka Koo, Sabine Ehrt, Helen Wainwright, Linda-Gail Bekker, Gilla Kaplan, Carl Nathan, Ira Tabas, David G. Russell

**Affiliations:** 1 Division of Molecular Medicine, Department of Medicine, Columbia University, New York, New York, United States of America; 2 Microbiology and Immunology, College of Veterinary Medicine, Cornell University, Ithaca, New York, United States of America; 3 Department of Microbiology and Immunology, Weill Cornell Medical College, Cornell University, New York, New York, United States of America; 4 Division of Anatomical Pathology, UCT Faculty of Health Sciences, Cape Town, South Africa; 5 The Desmond Tutu HIV Centre, Institute of Infectious Disease and Molecular Medicine, Department of Medicine, University of Cape Town, Cape Town, South Africa; 6 Laboratory of Mycobacterial Immunity and Pathogenesis, PHRI Center at the University of Medicine and Dentistry of New Jersey, Newark, New Jersey, United States of America; 7 Department of Medicine, Department of Pathology and Cell Biology, and Physiology and Cellular Biophysics, Columbia University, New York, New York, United States of America; 8 Microbiology and Immunology, College of Veterinary Medicine, Cornell University, Ithaca, New York, United States of America; Institute of Infectious Diseases and Molecular Medicine, South Africa

## Abstract

**Background:**

The endoplasmic reticulum (ER) stress pathway known as the Unfolded Protein Response (UPR) is an adaptive survival pathway that protects cells from the buildup of misfolded proteins, but under certain circumstances it can lead to apoptosis. ER stress has been causally associated with macrophage apoptosis in advanced atherosclerosis of mice and humans. Because atherosclerosis shares certain features with tuberculosis (TB) with regard to lesional macrophage accumulation, foam cell formation, and apoptosis, we investigated if the ER stress pathway is activated during TB infection.

**Principal Findings:**

Here we show that ER stress markers such as C/EBP homologous protein (CHOP; also known as GADD153), phosphorylated inositol-requiring enzyme 1 alpha (Ire1α) and eukaryotic initiation factor 2 alpha (eIF2α), and activating transcription factor 3 (ATF3) are expressed in macrophage-rich areas of granulomas in lungs of mice infected with virulent *Mycobacterium tuberculosis (Mtb)*. These areas were also positive for numerous apoptotic cells as assayed by TUNEL. Microarray analysis of human caseous TB granulomas isolated by laser capture microdissection reveal that 73% of genes involved in the UPR are upregulated at the mRNA transcript level. The expression of two ER stress markers, ATF3 and CHOP, were also increased in macrophages of human TB granulomas when assayed by immunohistochemistry. CHOP has been causally associated with ER stress-induced macrophage apoptosis. We found that apoptosis was more abundant in granulomas as compared to non-granulomatous tissue isolated from patients with pulmonary TB, and apoptosis correlated with CHOP expression in areas surrounding the centralized areas of caseation.

**Conclusions:**

In summary, ER stress is induced in macrophages of TB granulomas in areas where apoptotic cells accumulate in mice and humans. Although macrophage apoptosis is generally thought to be beneficial in initially protecting the host from *Mtb* infection, death of infected macrophages in advanced granulomas might favor dissemination of the bacteria. Therefore future work is needed to determine if ER-stress is causative for apoptosis and plays a role in the host response to infection.

## Introduction

Induction of the endoplasmic reticulum (ER) stress pathway known as the unfolded protein response (UPR) is activated in a number of disease processes such as neurodegenerative diseases, obesity, diabetes, atherosclerosis, heart disease, cancer, and viral infection [1]–[3]. The UPR is an adaptive survival pathway that becomes activated by accumulation of misfolded proteins in response to a variety of cellular insults that result in ER stress [4]. However, if ER stress is prolonged, or is combined with additional insults, this pathway can lead to cell apoptosis. ER stress-induced apoptosis is highly dependent on the upregulation of the UPR-inducible transcription factor CHOP (C/EBP homologous protein also known as GADD153). CHOP-deficiency has been shown to protect mice from cholestasis-induced liver fibrosis by reducing hepatocyte apoptosis [5]. Mice deficient in CHOP are also markedly protected from pancreatic β-cell apoptosis and diabetes [6]. Recently, CHOP deficiency was also found to protect from ER stress-induced apoptosis in cultured macrophages *in vitro*, and macrophage apoptosis was markedly reduced in advanced atherosclerotic lesions of *Chop*-deficient mice [7]–[9].

Both atherosclerotic lesions and tuberculous granulomas accumulate apoptotic macrophages and macrophage foam cells [10]–[13]. In atherosclerosis, the foamy appearance is caused by the accumulation of cholesterol ester lipid droplets [14]. Mycobacteria and their derived lipids such as oxygenated mycolic acids from virulent *Mycobacterium tuberculosis (Mtb)* or *Mycobacterium bovis* Bacillus Calmette-Guérin (BCG) induce a foam cell phenotype *in vitro*, and cholesterol ester accumulation occurs in *Mtb*-infected mouse lungs [15]–[18]. Importantly, recent work has shown that the primary lipids that accumulate in the caseum of a TB granuloma are unesterified cholesterol, cholesterol ester, and triacylglycerol supporting a concept that mycobacterial infection also causes the accumulation of host-derived lipids [19], [20]. Host-derived lipids such as 7-ketocholesterol [21] , saturated fatty acids [22], and unesterified cholesterol accumulation from the uptake of modified, aggregated, and remnant lipoproteins are known inducers of ER stress and contribute to macrophage apoptosis *in vitro* [7], [8], [23], [24]. Because ER stress has been causally associated with macrophage apoptosis in advanced atherosclerosis, and macrophage apoptosis is also associated with TB, we tested the hypothesis that ER stress may also be induced in macrophages of granulomas during *Mtb* infection.

## Results

### ER stress is induced in macrophages of granulomas during *Mtb* infection in mice

**Figure 1:**
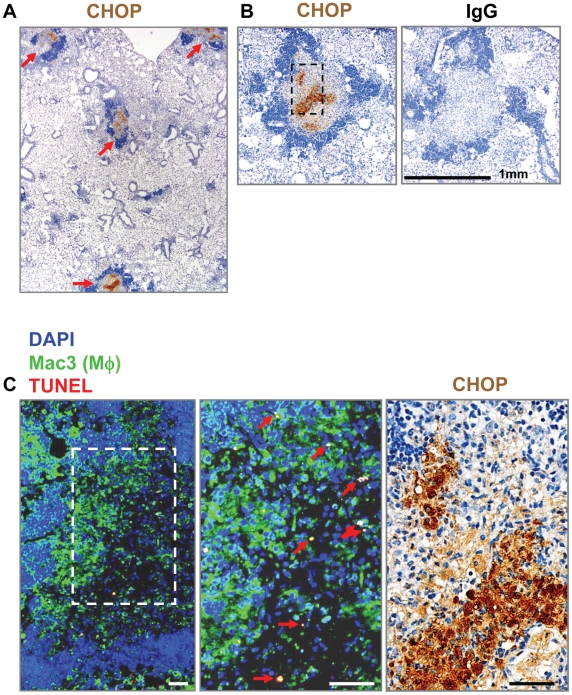
Apoptotic macrophages in CHOP-positive regions of *Mtb*-infected mouse lung. Mice were infected by aerosol with *Mtb*. Eight weeks later lungs were fixed and embedded in paraffin. Granulomas are identified in the center of each image and have rings of lymphocytes (dark blue) surrounding a central macrophage-rich core. **A and B.** Immunohistochemistry was performed using an antibody against CHOP and a normal IgG control. Slides were counter-stained with hematoxylin. **A.** Low power image of CHOP staining (brown) in several granulomas present (4x magnification, red arrows). **B.** IgG and CHOP staining (10x magnification, bar represents 1 mm). **C.** A serial section was stained for macrophages using an anti-Mac-3 antibody (green), and for apoptotic cells by TUNEL (red). Nuclei were stained using DAPI. Left and middle panels (20x and 40x magnification respectively) are merged images of Mac3, TUNEL, and DAPI stain. Black and white dashed boxes indicate corresponding areas of the granuloma of adjacent sections. Red arrows indicate TUNEL positive cells that were also positive for Mac3 and DAPI. Far right panel is the same area in a serial section, also shown in A and B, stained for CHOP by immunohistochemistry (40x magnification, bar represents 100 µm).

To examine if ER stress is induced during TB infection, we infected five 8 week old female C57BL6 mice by aerosol with *Mtb* strain H37Rv. Eight weeks postinfection, lungs were collected, sectioned, and stained by immunohistochemistry for various ER stress markers. This model is considered to be an early model of granuloma formation as lesions were identified by areas of consolidating pneumonitis without surrounding conspicuous fibrous capsules or centralized necrosis. Granulomas were characterized histologically by an abundance of lymphocytes and other cell types surrounding a macrophage-rich core. We found that the ER stress marker CHOP (C/EBP homologous protein also known as GADD153) was expressed in the majority of the granulomas of all five *Mtb*-infected mice examined. A representative image is shown in **Figure 1A**. The CHOP-positive staining occurred in the center of the granuloma where macrophages typically reside. Staining was not observed in the lymphocyte-rich area of the granuloma or when we stained a serial section with the normal rabbit IgG control antibody **(** **Figure 1B** **)**. To test for macrophage co-localization, we stained a serial sections using the macrophage marker Mac-3 **(** **Figure 1C, left panel** **)**. Corresponding boxed areas of **Figure 1B** **(upper panel)** and **Figure 1C** show that the CHOP-positive area was abundant in Mac3-positive macrophages. Some areas with the highest Mac-3 staining had relatively low levels of CHOP staining indicating that not all macrophages are expressing CHOP, or expressing very low levels. CHOP is a pro-apoptotic ER stress factor that is necessary for ER stress-induced apoptosis under many types of conditions. Recent data suggest that macrophage apoptosis may play a protective role in the host response to infection [13]. We therefore assayed for apoptosis by TUNEL analysis. We co-stained the Mac-3 stained sections by TUNEL to identify apoptotic cells and DAPI to confirm that the TUNEL stain was specific for DNA. We found numerous TUNEL-positive cells that were both Mac3 and DAPI-positive **(** **Figure 1C** **, middle panel)**. Moreover, TUNEL positive apoptotic cells (indicated by the red arrows) were abundant in the Mac-3 and CHOP-positive areas of the corresponding boxes **(** **Figure 1C, right panel** **)**.

**Figure 2:**
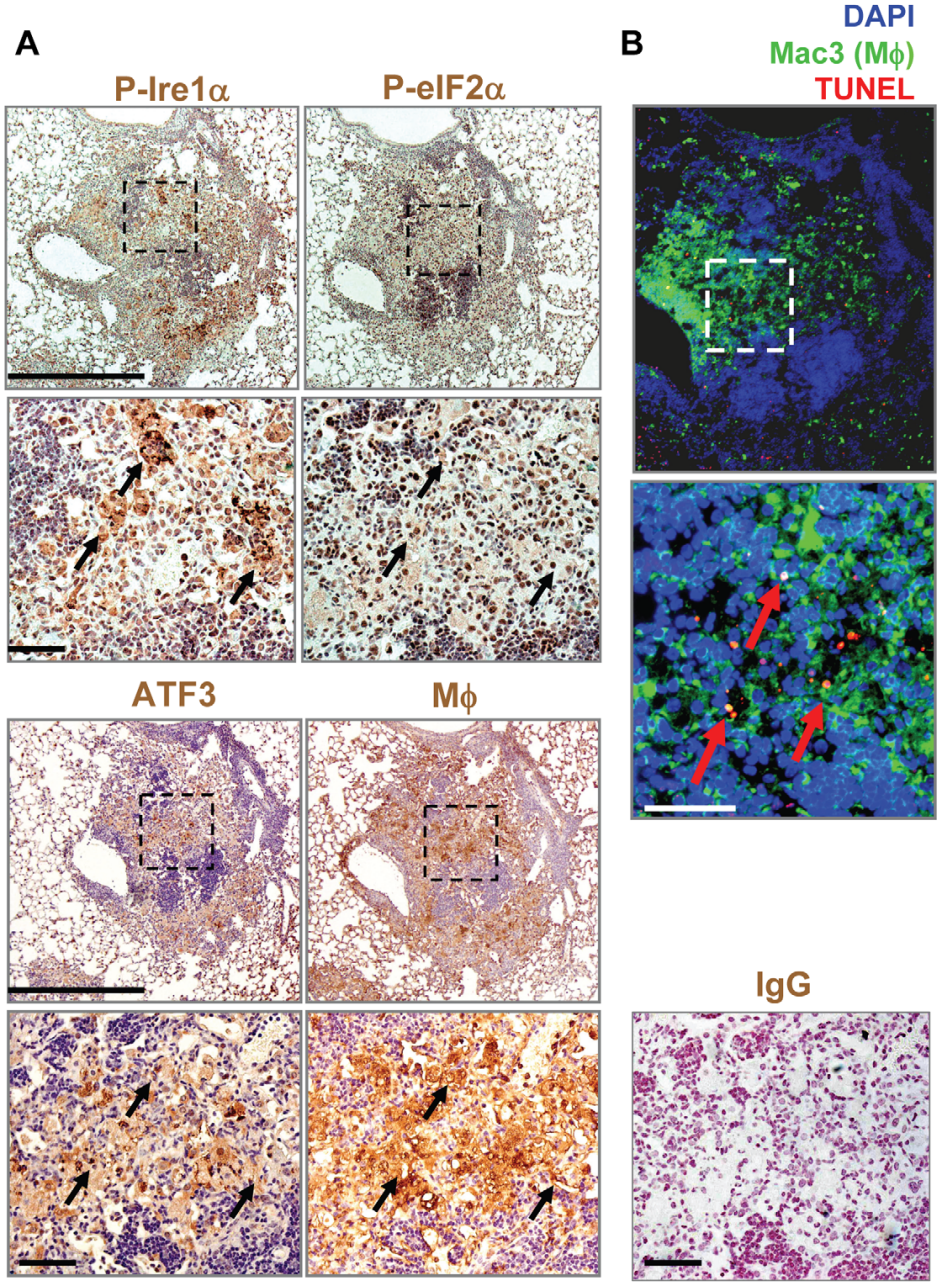
UPR markers are induced in macrophages residing in granulomas during *Mtb* infection in mice. Mice were infected as described in Figure 1. **A and B.** Immunohistochemistry was performed on lung sections using an antibody against Ser^724^ Ire1α (P- Ire1α), Ser^51^ eIF2α (P- eIF2α), ATF3, the macrophage (Mφ) marker AIA31240, and normal rabbit IgG. Slides were counter-stained with hematoxylin (upper panels, magnification 10x, bar represents 1 mm; lower panels, magnification 40x, bar represents 100 µm). **B.** A serial section was stained for macrophages using an anti-Mac-3 antibody (green), and for apoptotic cells by TUNEL (red). Nuclei were stained using DAPI (bar represents 100 µm). Dashed boxes in A and B indicate similar areas of the granuloma in adjacent sections that were enlarged in the corresponding images for clarity. The black arrows represent similar areas in the serial sections that are positive for all four markers. Red arrows indicate cells that were positive for TUNEL and the macrophage marker Mac-3.

We also tested for other markers of the ER stress pathway. Sections were stained by immunohistochemistry with antibodies that recognize phosphorylated Ire-1α, phosphorylated eIF2α, or the transcription factor ATF3. All were readily detected in the granulomas of *Mtb*-infected mice and colocalized with areas that were positive for the macrophage-specific antibodies AIA31240 and Mac 3 (corresponding dotted boxes, **Figure 2A and B** **)**. Staining was not observed with an IgG control antibody. Similar to Figure 1, we found many cells that costained with TUNEL, Mac3, and DAPI (red arrows) in areas that were also positive for our ER stress markers ATF3, phospho-Ire-1α, and phospho-eIF2α (corresponding dotted boxes, **Figure 2A and B**). These results indicate that several markers associated with the ER stress pathway are induced in macrophages of granulomas from *Mtb*-infected mice.

### ER stress is induced in granulomas during *Mtb* infection in humans

**Figure 3:**
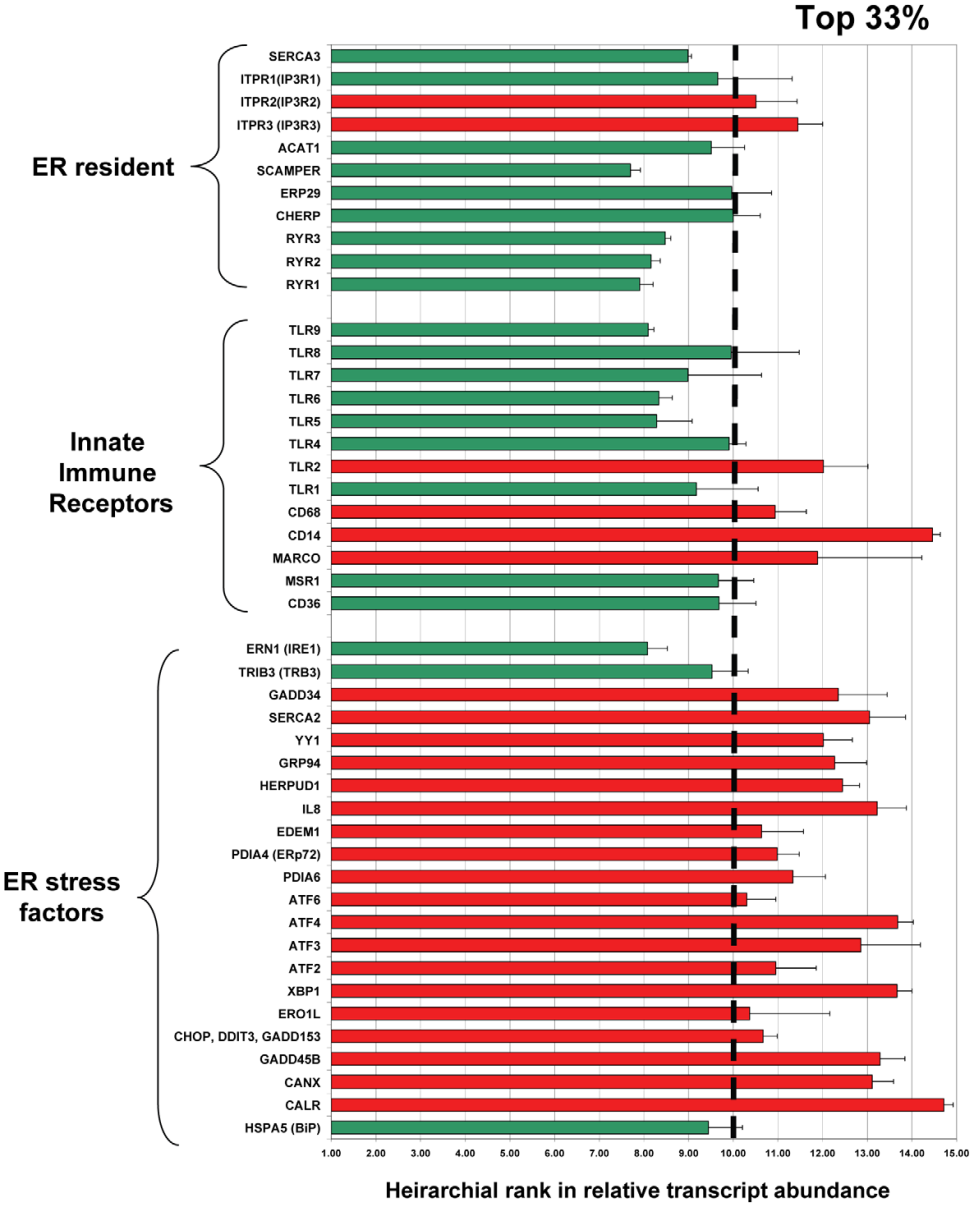
ER stress-induced genes are upregulated in human TB granulomas. RNA isolated from caseous granulomas by laser capture microdissection from 3 TB patients was subjected to microarray analysis. All genes in the database were ranked and a hierarchical list of each gene in relative transcript abundance was created. Shown is a comparison of ER resident genes that that have not been shown to be regulated by ER stress, common innate immune receptors such as scavenger receptors, TLRs, and macrophage markers, and genes known to participate in the Unfolded Protein Response or ER stress pathway. Error bars represent the standard deviation of caseous granulomas from three independent patients compared in the microarray. Genes represented by a red bars had a ranking above 10 and were represented in the top third of all genes in relative transcript abundance, and the genes represented by green bars fell below that threshold.

Our data showed that ER stress was induced in mouse granulomas during *Mtb* infection. We therefore tested if the ER stress pathway was also activated in human granulomas from the lungs of TB patients. Previously we had performed genome-wide microarray analysis on caseous granulomas from three independent lung tissue samples to explore which genes were upregulated and highly expressed in response to *Mtb* infection [20]. Caseous granulomas obtained from lung resection cases were isolated by laser capture microdissection (LCM) and captured RNA was amplified by PCR and hybridized on a GeneChip® Human X3P array. Because TB granulomas have markedly different cell types than that of non-infected lung parenchymal tissue, an arbitrary hierarchical scale was assigned to rank the genes from highest to lowest transcript abundance [20]. A ranking of greater than 10.00 is considered high for gene expression and represents approximately the top third of genes in relative transcript abundance. We mined the database for genes involved in the ER stress response and compared them with the expression of genes involved in innate immunity (TLRs and scavenger receptors) and resident ER proteins that are not regulated by ER stress. As shown in **Figure 3**, the classic ER stress-induced proteins including protein folding chaperones such as calreticulin (CALR), calnexin (CANX), heat shock proteins, and transcription factors such as GADD153 (CHOP), XBP1, ATF3, and ATF4, ranked among the most abundant transcripts in this database. Approximately 85% of the ER stress-regulated genes had a relative transcript abundance ranking over 10 (indicated in red). In contrast, the types A and B scavenger receptors SRA (MSR) and CD36, the majority of the toll-like receptors (TLRs), and many of the ER resident proteins that are not induced by ER stress were not as highly expressed (indicated in green).

A list of genes that were differentially regulated between the caseous human pulmonary TB granuloma and the control, which was uninvolved lung parenchyma from TB patients, was also generated [20]. Shown in **Table 1** is a list of the same genes shown in **Figure 3** that exhibited either an increase in transcript level over control, or no change. We found that 73% of the ER stress-regulated genes were increased in expression level over control, while only 18% of the ER resident genes not involved in ER stress were increased. We also found that 46% of the genes involved in innate immunity had increased expression. These results suggest that the UPR is induced in human tuberculosis at the mRNA transcript level.

**Table 1:** Genes involved in ER function, ER stress, and Innate Immunity.

ER RESIDENT	Fold increase (caseum vs. control, P<0.05)
RYR1	no change
RYR2	no change
RYR3	no change
CHERP	no change
ERP29	8.2
SCAMPER	no change
ACAT1	no change
ITPR3 (IP3R3)	no change
ITPR2(IP3R2)	26.8
ITPR1(IP3R1)	no change
SERCA3 (ATP2A3)	no change
**INNATE IMMUNE MARKERS**	
CD36	no change
MSR1	81.5
MARCO	106.2
CD14	399.6
CD68	no change
TLR1	57.4
TLR2	78.5
TLR4	20.1
TLR5	no change
TLR6	no change
TLR7	no change
TLR8	no change
TLR9	no change
**ER STRESS MARKERS**	
HSPA5 (BiP)	no change
CALR	13.3
CANX	16.9
GADD45B	no change
CHOP, DDIT3, GADD153	20.0
ERO1L	54.7
XBP1	22.5
ATF2	20.1
ATF3	no change
ATF4	no change
ATF6	33.8
PDIA6	65.8
PDIA4 (ERp72)	80.3
EDEM1	8.0
IL8	162.6
HERPUD1	43.0
GRP94	72.6
YY1	205.7
SERCA2 (ATP2A2)	206.1
GADD34 (PPP1R15A)	115.7
TRIB3 (TRB3)	no change
ERN1 (IRE1)	no change

Gene expression profiles were derived from three independent human caseous TB granulomas and averaged, then compared to that of uninvolved lung parenchyma. Shown is the fold upregulation as compared to the control (*P*<0.05). All genes listed under no change did not reach statistical significance.

### Induction of the ER stress markers CHOP and ATF3 occur in macrophages of human granulomas

**Figure 4:**
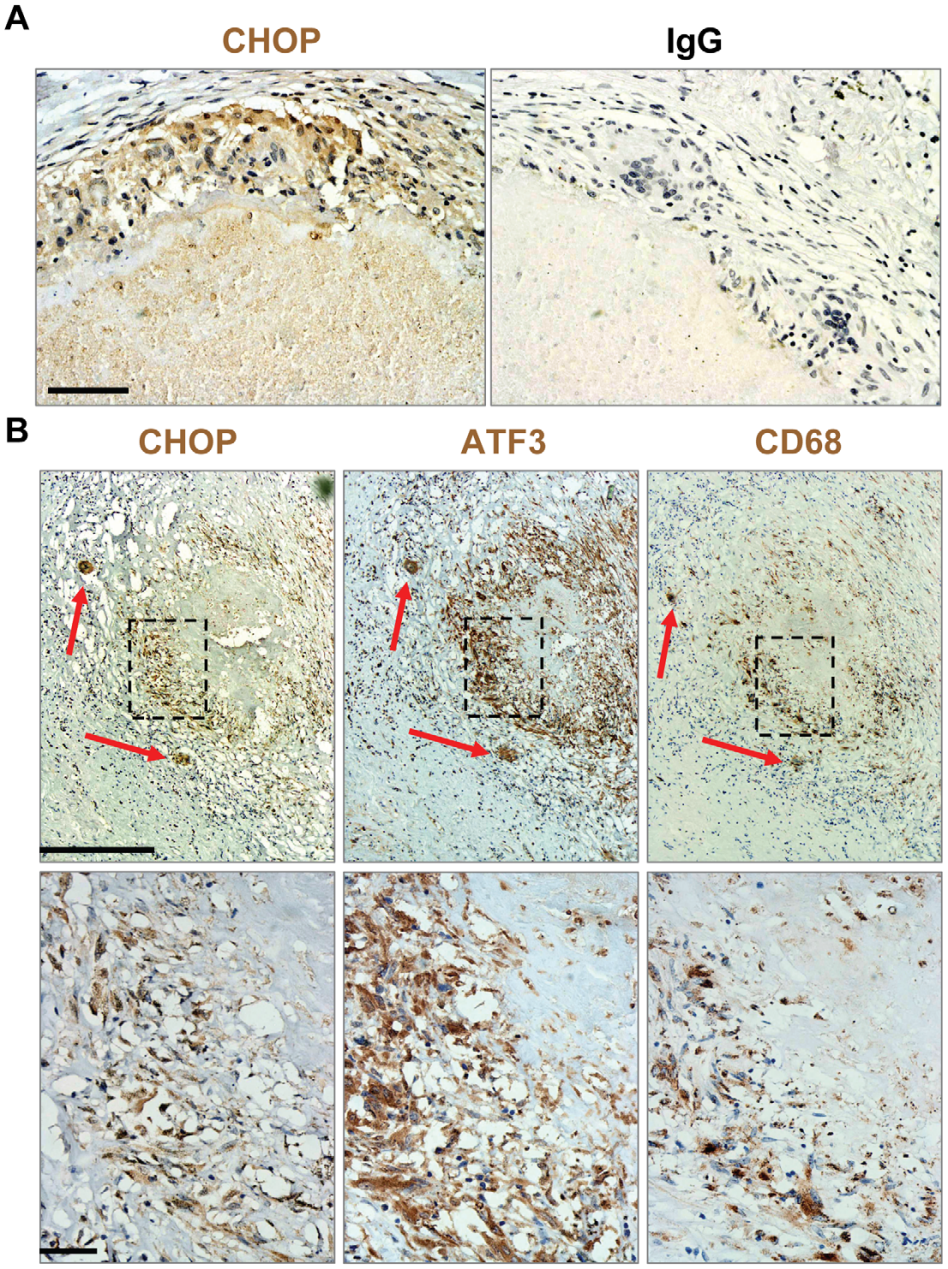
Induction of ER stress markers in human caseous TB granulomas. **A and B.** Lung sections from TB patients containing granulomas were stained by immunohistochemistry using an antibody against CHOP, normal IgG control, ATF3, and the macrophage marker CD68. Slides were counter-stained with hematoxylin. **A.** CHOP staining (brown), but not the IgG control, is seen around the central area of caseation (bar represents 100 µm). **B.** Low and high power magnification shows CHOP staining in areas that are also positive for ATF3 and CD68 (hatched box). The bar in the upper panel represents 1 mm while the bar in the lower panel represents 100 µm. Also shown are multinucleated giant cells surrounding the granuloma also positive for CHOP, ATF3, and CD68 (red arrows).

To determine if ER stress markers could also be detected at the protein level, we performed immunohistochemistry on granulomatous tissue collected from lung resections of 3 patients with TB. In all cases CHOP was highly expressed around the edges of the caseous granuloma, with some staining extending into the centralized area of caseation **(** **Figure 4A** **)**. In contrast, no staining was observed using the rabbit IgG control antibody. We also stained serial sections of these lung samples for ATF3 and the macrophage marker CD68. Again, in all cases, the CHOP-positive areas (left panels) were also positive for ATF3 (middle panels) and CD68 (far right panels) (**Figure 4B**). Lower magnification revealed the entire granuloma and showed a very similar staining pattern between CHOP, ATF3, and CD68 (hatched box), as well as co-staining in multinucleated giant cells surrounding the granuloma (long red arrows). We also found numerous cells that expressed ATF3 and CHOP that were not CD68 positive indicating that other cell types involved in granuloma formation may also be undergoing ER stress. These results suggest that ER stress is induced in macrophages and potentially other cell types during *Mtb* infection in humans.

### Induction of apoptosis in CHOP-positive areas of human granulomas

**Figure 5:**
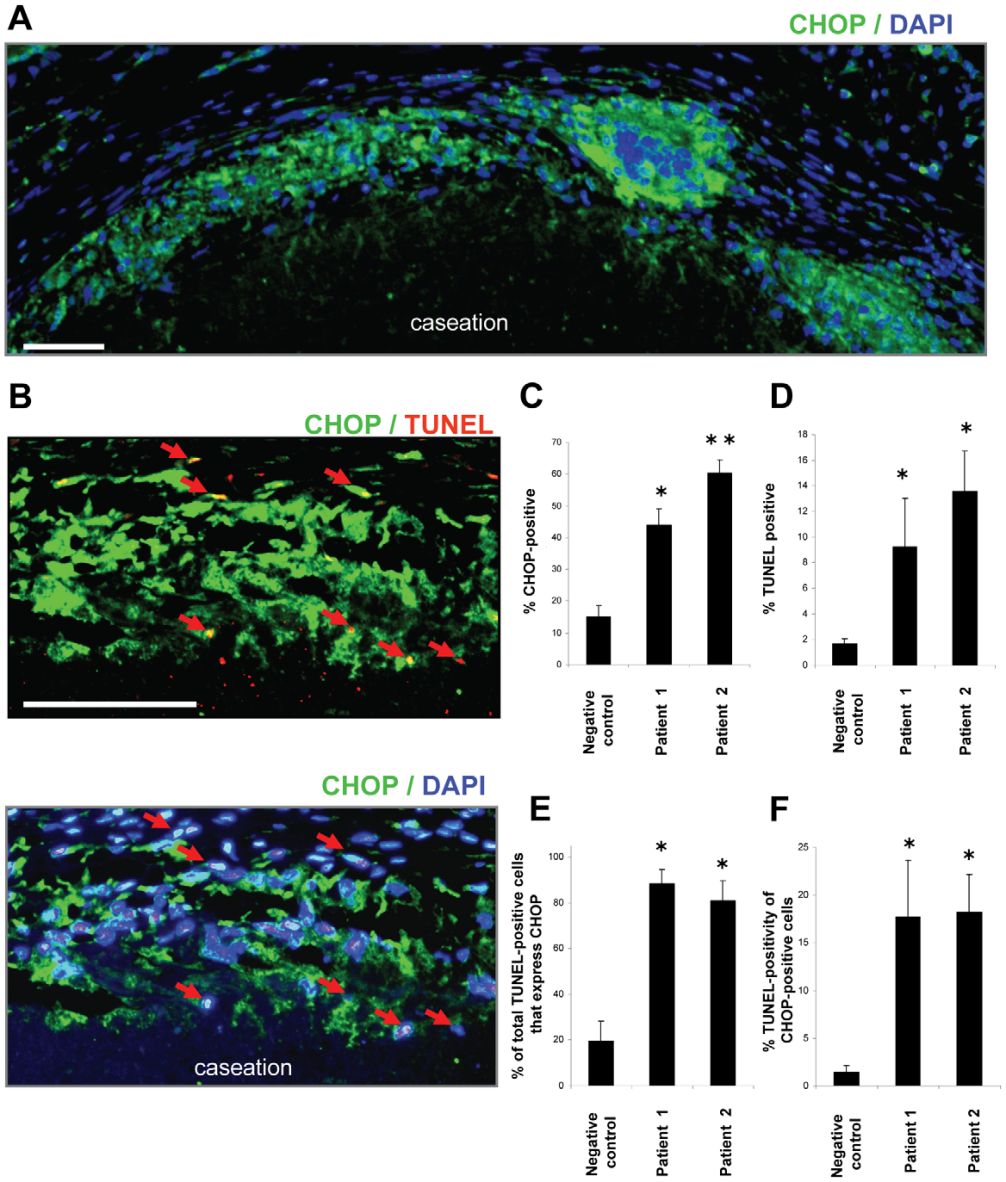
Increased apoptosis in CHOP-positive regions of human TB granulomas. Lung sections from TB patients containing granulomas were stained for CHOP (green), TUNEL (red), and nuclei (DAPI; blue). Patients 1 and 2 were positive for granulomas and the negative control was uninvolved lung parenchyma from a TB patient. **A.** CHOP staining (green) is seen around the central area of caseation (bar represents 100 µm). **B.** High power magnification shows TUNEL staining in areas that are also positive for CHOP and the DNA stain DAPI (red arrows, bar represents 100 µm). **C.** Quantification of the percent of CHOP-positive cells from 4 random fields of view. The number of CHOP-positive cells were expressed as a percent of total DAPI-positive cells in each field (n = 4 fields/patient). **D.** Quantification of the percent of TUNEL-positive cells from 4 random fields of view. The number of cells positive for both TUNEL and DAPI were expressed as a percent of total DAPI-positive cells in each field (n = 4 fields/patient). **E.** Quantification of the percent of total TUNEL-positive cells that express CHOP. The number of cells positive for TUNEL, DAPI, and CHOP were expressed as a percent of total number of TUNEL-positive cells in each field (n = 4 fields/patient). **F.** Quantification of the percent TUNEL-positivity in the CHOP expressing population. The number of cells positive for TUNEL, DAPI, and CHOP were expressed as a percent of total number of CHOP-positive cells in each field (n = 4 fields/patient). Differences between values with symbols and no symbols, or between values with different symbols, are statistically significant by ANOVA followed by post-hoc Student-Newman-Keuls test (*P*<0.05).

We next assayed for apoptosis in the CHOP-positive areas of human granulomas. Lung sections taken from the three TB patients were stained for CHOP, DAPI, and TUNEL. Two of the specimens contained granulomas and the third did not contain granulomatous tissue and was used as a negative control. Similar to the data in Figure 4, CHOP staining was most abundant surrounding the centralized areas of caseation in the granuloma-positive tissues **(** **Figure 5A** **)**. When the same sections were analyzed for apoptosis, we observed numerous TUNEL-positive cells that were also positive for the DNA stain (DAPI) within the CHOP-positive region surrounding the centralized area of necrosis **(** **Figure 5B** **)**. When the number of CHOP-positive cells was quantified and expressed as a percent of total cells that stained with DAPI, we found a significant increase in the percent of CHOP-expressing cells in the granuloma tissue versus the control patient **(** **Figure 5C** **)**. A corresponding increase in the percent of TUNEL-positive cells was similarly observed **(** **Figure 5D** **)**. Moreover, over 80% of the TUNEL-positive cells were also CHOP-positive in the granulomatous tissue of both patients, whereas less than 20% were CHOP-positive in the control patient **(** **Figure 5E** **)**. When we quantified apoptosis specifically in the CHOP-expressing population of cells, we observed a significant increase in apoptosis in the granuloma tissue versus the control **(** **Figure 5F** **)**. These results suggest that apoptosis correlates with CHOP expression in the granulomatous tissue of TB patients.

### ER stress enhances *Mycobacterium bovis* bacillus Calmette-Guérin (BCG)-induced apoptosis in cultured macrophages

**Figure 6:**
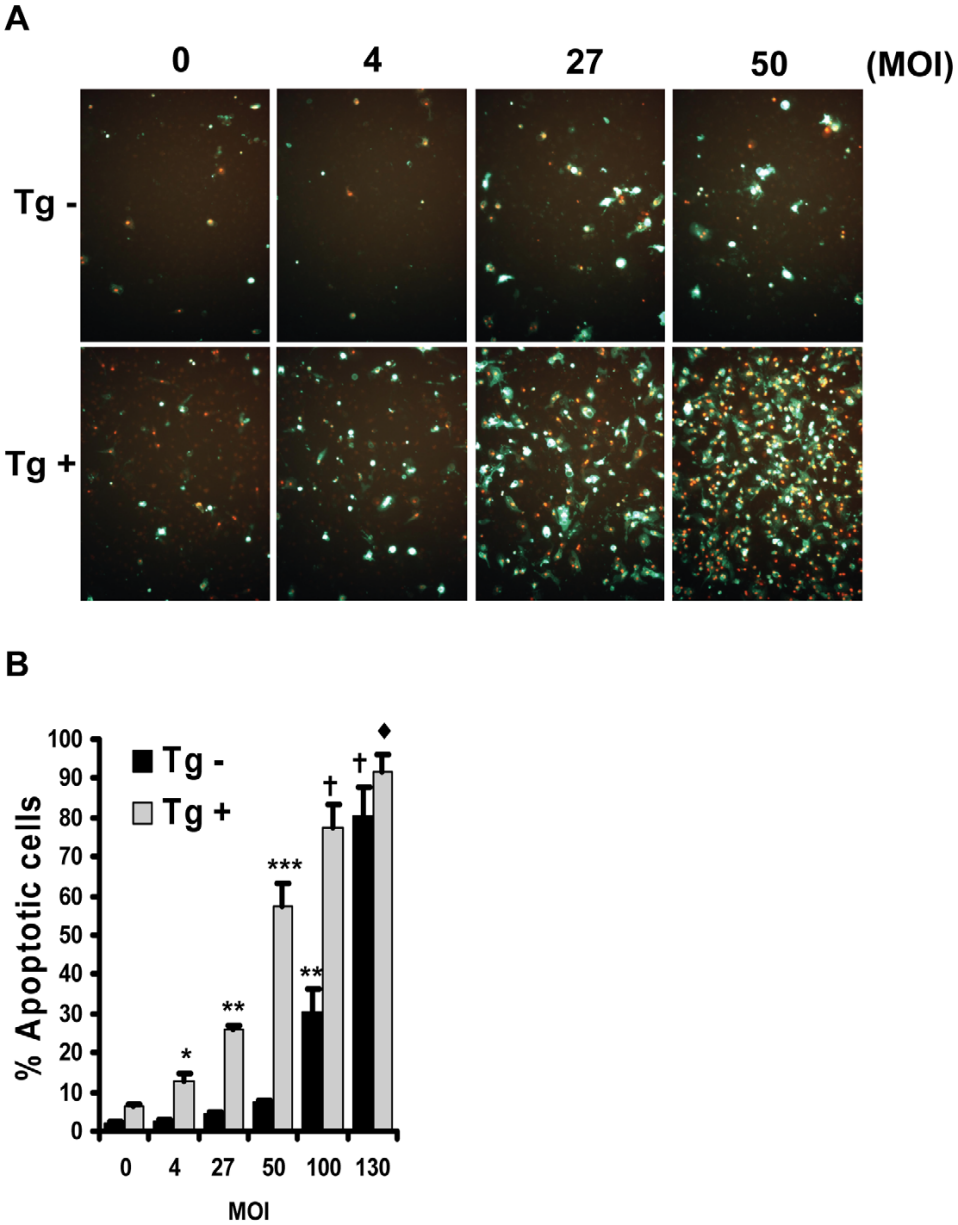
BCG-induced macrophage death is enhanced by thapsigargin. **A.** Murine peritoneal macrophages were infected with the indicated MOI of BCG in the presence or absence of 0.5 µM thapsigargin (an ER stressor). After 24 hours the macrophages were analyzed for apoptosis, and the data are expressed as the percent of total cells that stained with annexin V and propidium iodide (mean ± SEM; n = 4). Representative fluorescent images are shown. The presence of symbols or differing symbols indicate *P*<0.05; identical symbols or the absence of symbols indicate differences that are not significant by ANOVA post-hoc Student-Newman-Keuls test.

As shown in Figure 1, CHOP is induced in macrophages of *Mtb*-infected mice. Although CHOP expression has been shown to be necessary for ER stress-induced apoptosis, it is often not sufficient for inducing apoptosis [7], [25]. Rather, one or more additional cell insults are required in combination with UPR-CHOP activation to trigger apoptosis, perhaps to ensure that macrophage apoptosis only occurs when cell recovery is unlikely. We have recently discovered a multi-hit mechanism of macrophage apoptosis that involves the combination of pattern recognition receptor (PRR) engagement by ligands for the types A and B scavenger receptors (SRA and CD36) and ER stress [26]–[28]. Our data showing that ER stress is correlated with macrophage apoptosis in granulomas during *Mtb* infection suggest that ER stress may promote macrophage apoptosis during mycobacterial infection. To test this hypothesis, we examined if the addition of an ER stress-inducing agent could enhance macrophage apoptosis induced by the mycobacteria BCG**.** BCG was chosen based on previous work that has shown the bacilli to induce macrophage apoptosis and to be a known agonist of PRRs. Moreover, mycobacteria and their products, including BCG, have been shown to induce apoptosis and signal through multiple PRRs including SRA, CD36, MARCO, TLR2 and 4 [29]–[35]. Thapsigargin is an ER stress-inducing agent that, when given at a low dose, does not induce apoptosis by itself **(** **Figure 6** **)**. Likewise, we found that infection of murine peritoneal macrophages with BCG at a low multiplicity of infection (MOI <10) also resulted in low levels of apoptosis. However, when BCG was combined with thapsigargin, a synergistic increase in apoptosis was observed even when given at very low levels (MOI  = 4). These results suggest that BCG can synergistically trigger apoptosis when combined with an ER stress-inducing agent.

## Discussion

*Mtb* gains entry and survives in the host by infecting macrophages and suppressing macrophage death [36], [37]. In spite of the evidence that apoptosis can be suppressed by *Mtb*, macrophage apoptosis is induced during *Mtb* infection *in vitro*, and apoptotic macrophages are found inside granulomas of *Mtb*-infected lung [38], [39].

Based on the previous work in our laboratory on the role of ER stress in macrophage apoptosis in atherosclerosis, we set out to test the hypothesis that ER stress may also be induced in TB; a disease in which macrophage apoptosis has been hypothesized to be beneficial to the host. Our finding that ER stress is induced in macrophages residing in mouse and human lung granulomas during *Mtb* infection, notably where apoptotic cells accumulate, reveals a striking commonality to what we and others have observed in advanced atherosclerosis [9], [21], [40]. Similar to atherosclerosis, the exact cause of ER stress in TB is unknown. Some of these insults may arise from the accumulation of ER stress-inducing host-derived lipids such as 7-ketocholesterol [21], long chain saturated fatty acids [22], and unesterified cholesterol [8], which also contribute to macrophage apoptosis *in vitro* [24]. Indeed, one of the primary lipids that accumulate in the caseum of a TB granuloma is unesterified cholesterol [20], [41]. In addition to host-derived lipids, other potential causes of ER stress in granulomas that could promote apoptosis are mycobacteria themselves, peroxynitrite, or hypoxia, which are associated with mycobacterial infection [42]–[44]. For example, ESAT6, an antigen that is secreted by *Mtb* and inducer of macrophage apoptosis, was recently identified to activate the ER stress pathway [45].

Our cell culture data show that the addition of an ER stress-inducing agent synergizes with BCG to trigger apoptosis, whereas neither of the agents induced apoptosis on their own when given at low concentrations. We have recently shown that ER stress in combination with additional insults, for example when an ER stressor is combined with atherosclerosis-relevant PRR ligands that recognize either the type A scavenger receptor (SRA) in combination with TLR4, or the type B scavenger receptor (CD36) in combination with TLR2, a synergistic induction of macrophage apoptosis ensues [46]. Previous studies have revealed that mice deficient in CHOP or in the PRR's SRA, CD36, TLR4, or TLR2, are markedly protected from macrophage apoptosis by this two-hit pathway [9], [24], [27], [46]. *In vivo*, there are many endogenous inducers of ER stress, e.g., unesterified cholesterol from host-derived lipoproteins, oxysterols, and peroxynitrite that may coexist with various SRA, TLR4, CD36, and TLR2 ligands in granulomas, e.g., mycobacteria and their lipid products and oxidized-phospholipids [30], [31], [47]–[49]. Recently, it has been demonstrated that foam cell formation *in vitro* can be induced by the bacterial cell wall lipid trehalose dimycolate signaling through TLR2, which could explain both the lipid sequestration and the induction of programmed cell death [20], [31]. The combination of these events could therefore contribute to macrophage apoptosis in granulomas during TB infection. Although we were able to show that ER stress enhances BCG-induced macrophage apoptosis, future work is needed to establish causation of the ER stress pathway in macrophage apoptosis during mycobacterial infection. For example, studies using CHOP or PRR-deficient mice and human-derived macrophages would be valuable to determine if any differences exist in ER-stress-induced macrophage apoptosis when comparing virulent and less virulent strains of mycobacteria.

Both the mechanisms and functional consequences of macrophage apoptosis in TB have been the subject of intense investigation and debate. Apoptosis, but not necrosis of infected monocytes is coupled with killing of intracellular BCG [50], [51]. Although all mycobacteria are capable of inducing apoptosis to some degree, less virulent mycobacteria such as BCG and avirulent *Mtb* (H37Ra) are better inducers of macrophage apoptosis when directly compared to the more virulent mycobacteria such as *Mtb* (H37Rv)[11], [33], [37], [52]. From these data a hypothesis has emerged that mycobacterial pathogenicity is inversely correlated with the ability of macrophages to undergo apoptosis [13]. This hypothesis is supported by results that suggest two distinct advantages in promoting macrophage apoptosis during early mycobacterial infection. First, the increased bacterial killing that was observed in apoptotic macrophages in combination with clearance of apoptotic cells may prevent the spread of infection and lead to sterilization of the infected host [12], [39], [51], [53], [54]. Second, the uptake of infected apoptotic macrophages by dendritic cells (DCs) leads to the breakdown and processing of *Mtb* antigen [55], which is then presented via MHC class I to T-cells stimulating the adaptive immune response [55]. Two groups also showed that delivery of proapoptotic *Mtb* mutants are more effective at eliciting a host response to *Mtb* challenge [55], [56]. In late-stage infection, however, the consequence of cell death is likely to change. A late-stage tuberculosis granuloma in humans has little vascularization and a restricted supply of both macrophages and lymphocytes [57]. The induction of cell death in infected macrophages and the foamy macrophages that are abundant in late-stage disease will lead to the accumulation of caseum and the progression of pathology. *Mtb* takes advantage of this pathology, the liquefaction and cavitation of the granuloma, to release infectious bacilli into the airways. Therefore while apoptosis might be beneficial to the host at low bacterial numbers early in infection, it is more likely to be detrimental once the disease has progressed and fibrocaseous granulomas have formed [13], [41].

As our data correlate induction of ER stress and apoptosis with tuberculosis, the challenge at hand will be to causally connect our findings to the overall outcome of infection in patients.

## Materials and Methods

### Ethics Statement

For the human study all protocols were reviewed and approved by the ethical review boards at UMDNJ, Cornell University, and the University of Cape Town. Informed consent was obtained from all of patients on a consent form approved by all institutional IRBs. All mouse procedures performed in this study were reviewed and approved by the Institutional Animal Care and Use Committee of Weill Cornell Medical College (Animal protocol # 0807-768A).

### Reagents

Falcon tissue culture plastic was purchased from Fisher Scientific. Tissue culture media, cell culture reagents, and heat-inactivated fetal bovine serum (FBS) were from GIBCO. Thapsigargin (Tg), concanavalin A, DAPI, and Meyers hematoxylin were purchased from Sigma. Antibodies were purchased from the following sources: rabbit polyclonal antibody to CHOP, ATF3, and normal rabbit IgG control—Santa Cruz Biotechnology; Mac-3 antibody and biotinylated anti-rat IgG—BD Biosciences; mouse monoclonal antibody to actin—Chemicon; CD68 antibody—DAKO; antibody against Ser^724^-Ire1α, Ser^51^-eIf2α—Abcam; horseradish peroxidase-conjugated donkey anti-rabbit IgG secondary antibodies—Jackson ImmunoResearch; donkey anti-rabbit Alexa-488—Invitrogen/Molecular Probes.

### Mouse infection

Eight to ten weeks old female C57BL/6J mice were infected with *Mtb* using an aerosol chamber (Glas-Col Inc.). Animals were exposed to an aerosol produced by nebulizing 5 ml bacterial single cell suspension in PBS at ∼2×10^8^ bacilli/ml resulting in 175±98 colony forming units per lung as determined by plating homogenized lungs 24 h postinfection on enriched Middlebrook 7H11 agar plates. Eight weeks postinfection, the upper left lung lobe was fixed in 10% formalin and embedded in paraffin.

### Human tissue specimens

Human tuberculosis tissues were obtained and processed as previously described [20]. Human TB lung tissues were surgically excised from TB patients who had extensive lung cavitation and tissue degeneration. The patients who were not responding to canonical antibiotics underwent surgery [20].

### Laser Capture Microdissection

Tissue sections we processed as detailed previously [20].

### Microarray

Microarray was performed as described previously [20].

### Microarray data analysis

Microarray data were analyzed as described previously [20]. The complete genelist, together with a second list comparing granuloma tissue to uninvolved lung tissue, are accessible through GEO database, NCBI accession number GSE20050 [20]. The statistical significance of differences in gene expressions between uninvolved human lung parenchyma tissues and caseous TB lung granulomas was calculated using an unpaired t test with Benjamini-Hochberg correction. Results with a *P* value less than 0.05 was considered significant [20].

### Mouse peritoneal macrophages and bacteria

Peritoneal macrophages from adult female C57BL/6J mice and all mutant mice used in this study were harvested three days after i.p. injection of concanavalin A [7]. All macrophages were grown in full medium containing DMEM (25 mM glucose), 10% FBS, and 20% L-cell conditioned medium solution (GIBCO). The medium was replaced every 24 h until cells reached 90% confluency. One day before the experiment, the cells were dissociated and counted. Macrophages were plated on 48 well tissue culture plates at a density of 75,000 cells per well in the same medium. *Mycobacterium bovis* bacillus Calmette-Guérin (BCG Pasteur strain, American type Culture Collection) were grown to early log phase in Middlebrook 7H9 medium with ADN enrichment and 40 mM Sodium Pyruvate, and 0.05% Tween80 to prevent clumping. The bacteria were pelleted and resuspended in PBS and 0.05% Tween80, vortexed, and prepared as a single cell suspension by a series of low speed centrifugations (800rpm for 10 minutes). Macrophages were infected with BCG at increasing multiplicity of infection based on optical density (0.1OD_600_ = 5×10^7^ bacterial/ml). The input of bacteria was verified by plating in triplicate on 7H10 agar supplemented with OADC and colonies were counted two weeks later to obtain the multiplicity of infection (MOI).

### Antibody staining of mouse and human sections

Lungs were immersion-fixed in 10% neutral-buffered formalin overnight followed by embedding in paraffin. 7 µM sections on glass slides were deparaffinized in xylene and hydrated in water. Antigen retrieval was obtained by exposing the slides for 20 min to 1 mM EDTA, pH 8.0, in a steamer**.** The sections were incubated with the appropriate antibody overnight, and stained for immunofluorescence or immunohistochemistry. For immunohistochemistry, the sections were then treated with 3% H_2_O_2_ to inactivate the endogenous peroxidase. The sections were stained using the rabbit ABC staining system from Santa Cruz Biotechnology according to the manufacturer's protocol. The sections were then counterstained with Mayers Hematoxylin and examined by light microscopy.

For immunofluorescence assays, the sections were permeabilized with 0.1% Triton X-100 and then incubated with anti-Mac-3 antibody overnight, followed by incubation with a biotinylated anti-rat IgG, and a streptavidin-conjugated Alexa 488-labeled secondary antibody. Sections were then stained for TUNEL as described below, followed by counter staining with DAPI to identify nuclei.

### Apoptosis assays

For the *in vitro* experiments, apoptosis was assayed in cultured macrophages by staining with Alexa 488-conjugated Annexin V (green) and propidium iodide (PI; red), as described previously [7], [25]. The number of Annexin V- and PI-positive cells were counted and expressed as a percent of the total number of cells in at least 4 separate fields from duplicate wells. Representative fields (4–6 fields containing ∼1000 cells) were photographed. Apoptotic cells in tissues were labeled by TUNEL (TdT-mediated dUTP nick-end labeling) using the *in situ* cell death detection kit TMR-red (Roche Diagnostics) according to the manufacturer's protocol. Only TUNEL-positive cells that co-localized with DAPI-stained nuclei and Mac3 were counted as being positive. TUNEL and annexin staining were viewed using an Olympus IX-70 inverted fluorescent microscope equipped with an Olympus DP71 CCD camera. For TUNEL analysis, DAPI, TUNEL, and/or Mac3 images were merged using Photoshop analysis software (Adobe Systems). The number of cells positive for TUNEL, DAPI, and CHOP (human sections only) were counted from four fields obtained from each section.

### Statistics

Values are given as means ± S.E.M. unless noted otherwise in the figure legend. The number of replicates is noted in the figure legends. Absent error bars in the bar graphs signify S.E.M. values smaller than the graphic symbols. Comparison of mean values between groups was evaluated by an ANOVA followed by a post-hoc Student-Newman-Keuls test. For all statistical methods a *P* value less than 0.05 was considered significant.
